# Freezing tissues pre-homogenisation reduces degradation and yields improved quality RNA in the mouse lung

**DOI:** 10.1016/j.bbrep.2025.102332

**Published:** 2025-11-06

**Authors:** Khairunnisa Mohd Kamal, Ahmad Rohi Ghazali, Gayathri Thevi Selvarajah, Nurul Syakima Ab Mutalib, Nadiah Abu, Eng Wee Chua, Siti Fathiah Masre

**Affiliations:** a Biomedical Science Programme, Centre for Toxicology and Health Risk Studies (CORE), Faculty of Health Sciences, Universiti Kebangsaan Malaysia (UKM), Kuala Lumpur, 50300, Malaysia; b Department of Veterinary Clinical Studies, Faculty of Veterinary Medicine, Universiti Putra Malaysia (UPM), Serdang, 43400, Malaysia; c UKM Medical Molecular Biology Institute, Universiti Kebangsaan Malaysia (UKM), Cheras, Kuala Lumpur, 56000, Malaysia; d Centre for Drug and Herbal Development, Faculty of Pharmacy, Universiti Kebangsaan Malaysia (UKM), Kuala Lumpur, 50300, Malaysia

**Keywords:** RNA extraction, Homogenisation, Optimisation, Tissue samples, Frozen

## Abstract

Ribonucleic acid (RNA) extraction requires meticulous sample handling to ensure purity and integrity. Although a variety of commercial kits are available, along with optimised protocols, pre-extraction sample processing remains a challenging procedure, especially with tissue samples. In our brief report, we describe the beneficial impact of freezing tissues before homogenisation on the quality of RNA extraction. Lung tissues were freshly excised from mice and homogenised with or without prior quick freezing in a freezer. Then, RNA was extracted according to the protocol provided with a commercial column-based RNA extraction kit. RNA quality was analysed by UV absorbance and agarose gel electrophoresis. We found that the frozen tissues yielded better-quality, more intact RNA than the non-frozen tissues, possibly due to lower temperatures during homogenisation. “Smearing”, indicative of RNA degradation, was visible in some of the non-frozen samples. The extra quick-freezing step provides a simple and affordable method for preserving high-quality RNA, especially from tissue samples. Further comparisons can be made to determine whether the observed benefits extend to other tissue types or to quantitative polymerase chain reaction (qPCR) analysis in gene expression studies.

## Highlights

From: Freezing tissues pre-homogenisation reduces degradation and yields improved quality RNA.

• Pre-extraction of tissue samples requires a meticulous sample handling procedure
• Freezing tissues prior to homogenisation yields better quality and purity of RNA
• More intact RNA is observed in frozen tissues using gel electrophoresis
• The quick-freezing method benefits RNA extraction using an affordable and less time-consuming procedure

## Graphical abstract

**Figure:**
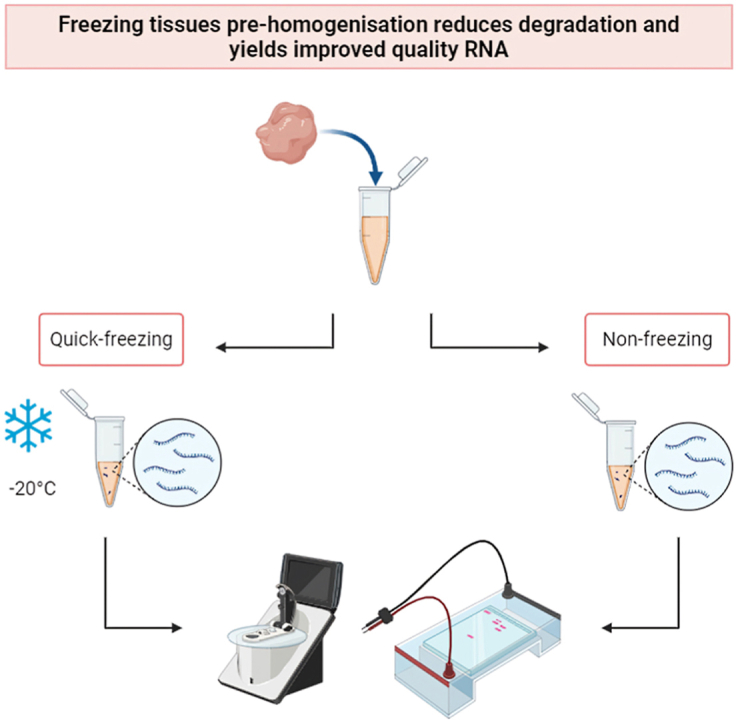

## 1 Introduction

**Fig. 1:**
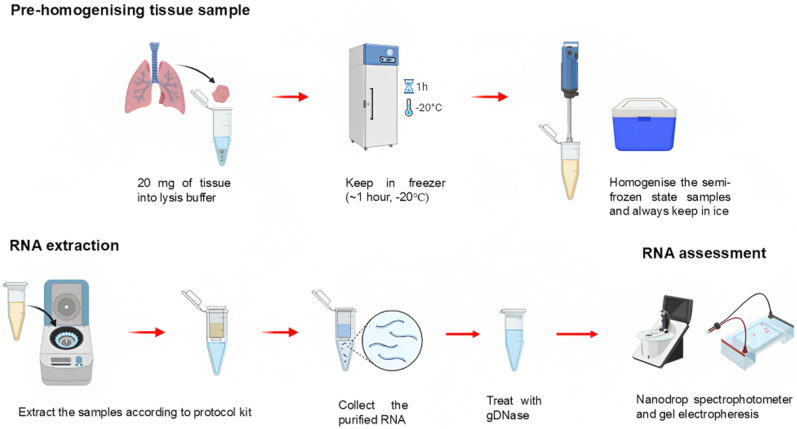
Illustration of pre-homogenising steps prior to RNA extraction. Created with Biorender.com.

Ribonucleic acid (RNA) is the crucial input material for gene expression studies [1]. However, the procedure to isolate intact, high-quality RNA, especially from tissue samples, is challenging and requires careful sample preparation in an RNase-free environment. RNA is heat-labile and readily degraded by ubiquitous RNases [2], prompting the adoption of two often-cited countermeasures: using freshly excised tissues and maintaining cold temperatures throughout sample processing. Recently, we found that freezing tissue samples before homogenisation could help offset the heat produced and boost RNA quality (Fig. 1). In this brief report, we examine the beneficial impact of the additional procedure that is not initially included in the manufacturer's protocol for a commercial column-based kit, by comparing the quality of RNA extracted from fresh lung tissues frozen in lysis buffer with that from non-frozen tissues using gel electrophoresis and spectrophotometer analysis.

## 2 Materials and methods

### 2.1 Animal work

The project was approved by the Universiti Kebangsaan Malaysia Animal Ethics Committee (FSK/2023/SITI FATHIAH/22-NOV./1384-DEC.-2023-NOV.-2026) and conducted in compliance with the ARRIVE guidelines. Lung tissues (20 mg) were dissected from female BALB/c mice, immediately immersed in 450 μL of lysis buffer, and divided into two groups, i.e. frozen and non-frozen (n = 4 each). The sample size in this study was determined using the same formula as an established study [3]:

n=DF/k+1,

where DF is the degree of freedom (between 10 and 20), and k is the number of groups to be applied in the study.

Using DF = 20 and k = 6, the sample size was calculated as n = 20/6 + 1 = 4.

For the frozen group, the tissue samples were immersed in lysis buffer and kept at −20 °C for about 1 h. Then, the samples were thawed for approximately 15 s before being homogenised on ice using a rotator homogeniser, Ultraturax T25 (IKA, Germany), for about 10 s, repeated two to three times until all the tissues were completely lysed. The non-frozen tissues were immersed in lysis buffer and placed on ice accordingly until the last sample was harvested, a process that took approximately 1 h. They were then immediately homogenised using a rotator homogeniser.

### 2.2 RNA extraction

RNA was extracted using the innuPREP RNA Mini Kit (IST Innuscreen, Denmark). The manufacturer's protocol was modified slightly, where the centrifugation temperature was lowered to 4 °C. Otherwise, the samples were processed according to the recommended protocol to yield RNA solutions.

### 2.3 Removing genomic DNA

Genomic DNA was removed from extracted RNA using the RapidOut DNA Removal kit (Thermo Scientific, USA). The final supernatant, containing pure RNA, was transferred into a new tube and stored at −80 °C until further use.

### 2.4 RNA quality assessment

For measuring concentrations and purity, 1 μL of RNA was placed on the pedestal of a NanoDrop 2000 spectrophotometer (Thermo Fisher Scientific, USA), and UV absorbance at 260 nm and 280 nm was recorded. Native 1 % agarose gel electrophoresis was also conducted to assess RNA integrity. A total of 200 ng of RNA was mixed with a loading dye (Vivantis, Malaysia), loaded into an agarose gel, and electrophoresed at 70 V for 45 min. Using the Chemiluminescent Molecular Gel Imager (Bio-Rad, USA), the separated RNA molecules were visualised. Well-defined bands with minimal “smearing” indicated high RNA quality.

### 2.5 Statistical analysis

Descriptive analysis and *t*-test were applied to analyse the concentration of RNA and the purity (A260/280 ratios) with a significant value at p < 0.05 using GraphPad Prism version 9.

## 3 Results and discussion

The RNA concentrations for the frozen and non-frozen samples were comparable (p > 0.05) (Table 1) with purity within the acceptable range, i.e. A260/280 ratios of 1.8–2.1 (Table 2).

**Table 1:** Statistical data of RNA concentrations and A260/280 ratio.

Sample	Mean ± SEM		p-value
	Concentration (ng/uL)	A260/280 ratio	
Frozen	163.0 ± 20.51	2.10 ± 0.0047	0.15
Non-frozen	214.6 ± 53.03	2.08 ± 0.0135

**Table 2:** Sample RNA concentrations measured by the spectrophotometer.

Sample	Concentration (ng/uL)	A260/280 ratio
Frozen 1	208.0	2.09
Frozen 2	134.3	2.11
Frozen 3	122.7	2.11
Frozen 4	187.0	2.10

Non-frozen 1	186.5	2.08
Non-frozen 2	77.5	2.13
Non-frozen 3	280.6	2.08
Non-frozen 4	313.6	2.07

**Fig. 2:**
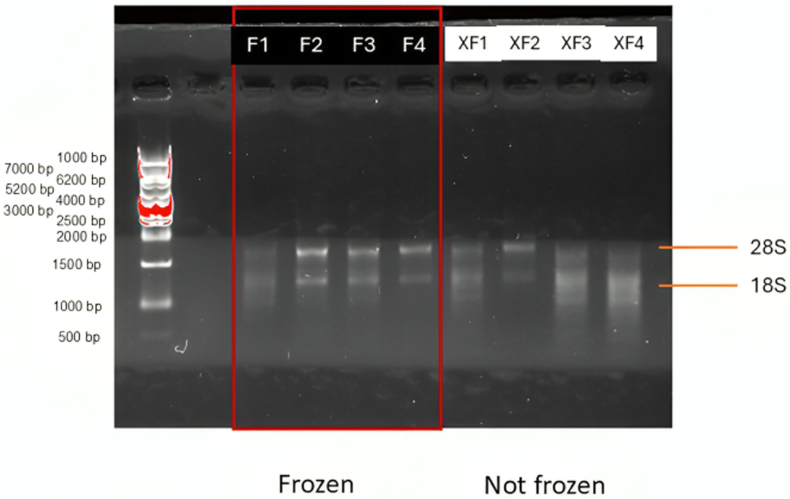
Agarose gel analysis of RNA extracted from tissue samples homogenised with or without prior quick freezing. Abbreviations: F- frozen; XF- non-frozen.

**Fig. 3:**
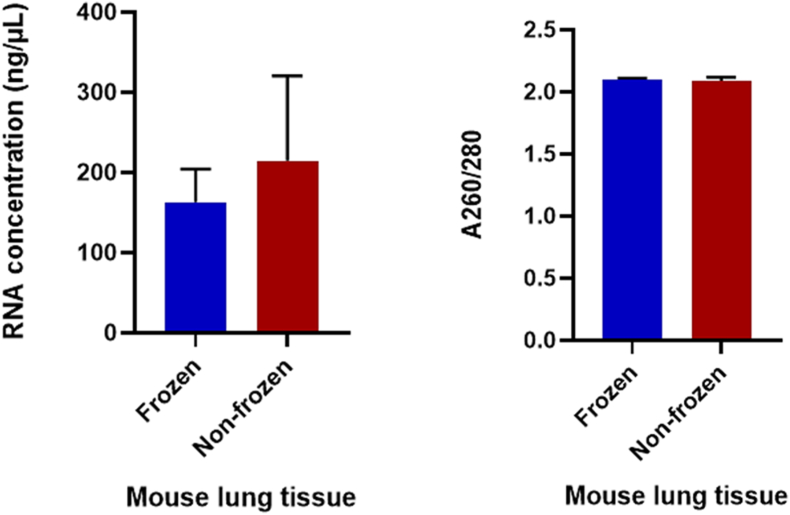
Analysis of RNA concentration and purity using a spectrophotometer for both frozen and non-frozen mouse lung tissue.

Despite no apparent differences in RNA concentrations, analysis by agarose gel electrophoresis revealed that frozen samples yielded better-quality, more intact RNA than non-frozen samples (Fig. 2). Some of the non-frozen samples (3 and 4) showed visible signs of degradation (“smearing”), possibly caused by heat produced during homogenisation. According to statistical data, RNA concentrations in frozen samples exhibited less variation and higher purity compared to those in non-frozen samples (Fig. 3).

Tissue homogenisation is an early procedure in isolating cellular components for further analysis. The main challenge in ensuring complete homogenisation is the excess heat generated during prolonged sample processing, which can degrade RNA [4]. Therefore, the procedure must be kept cold, for example by placing samples on ice, to minimise possible RNA degradation. Freezing tissues before homogenisation may further offset the heat produced and more effectively preserve RNA integrity and quality. The quick-freezing of samples immersed in lysis buffer was applied in our study to minimise the rise in sample temperature throughout homogenisation.

A similar (and related) approach is snap-freezing tissue samples in liquid nitrogen to preserve their integrity [5]. However, the extremely low temperature of liquid nitrogen renders it a hazardous substance that requires specialised equipment for storage and precautionary steps during handling. The simple method described in this study offers a convenient alternative to the use of liquid nitrogen.

A recent study by Poutoglidou et al. (2021) has also adopted the method of freezing tissue samples in a lysis buffer before homogenisation and successfully obtained high-quality, intact RNA [4]. Similarly, their studies also indicated that keeping tissue samples on ice or in an ice bath cannot completely prevent RNA degradation caused by heat produced during homogenisation [4]. The primary difference between the previous study and our study is that we froze tissues in a lysis buffer for 1 h, whereas Poutoglidou et al. used a specialised RNA-preserving solution, RNAlater-ICE, and stored their samples overnight. RNAlater-ICE prevents RNA degradation during thawing and homogenisation of frozen tissues. By not using chemical stabilisers such as RNAlater-ICE, we were able to directly evaluate the beneficial effect of pre-homogenisation freezing on RNA extraction quality in a short period of time. Moreover, the use of RNAlater-ICE has a few drawbacks – it adds costs and complications to the extraction process. Paradoxically, RNAlater has been reported to impair sample stability by rendering tissues harder and its high density requires high centrifugal forces to pellet samples [6]. It is also reported that storage of samples in RNAlater decreases cell lysis efficiency compared to normal glycerol [6].

The main limitation of our method is that freezing tissues before homogenisation is somewhat counterintuitive, as freeze-thawing is typically regarded as detrimental to RNA. Repeated or prolonged freeze-thawing of tissues can degrade RNA by causing the formation of ice crystals and activating RNases, as RNA is susceptible to physical disruption of cellular structures [7]. Additionally, we have not tested the effect of shorter freezing times (<1 h) on RNA quality and in different types of tissue, which could be explored in future studies. As the study primarily aims to observe the effects of temperature on tissue homogenisation in relation to RNA quality, it is limited to gel electrophoresis and concentration, with purity assessment only. Further analysis could benefit from this approach in the future. Several studies have suggested that immediate or rapid freezing in liquid nitrogen or on dry ice, followed promptly by RNA extraction, is required to preserve RNA integrity, particularly in soft tissues [8,9]. Therefore, while our method yielded high-quality RNA, further optimisation may benefit from reducing the freezing time to minimise potential degradation associated with the freeze-thaw process, as well as the involvement of different types of tissues and lysis buffers. This is because different tissue types may produce different outcomes, and different reagents in the lysis buffer could also affect the homogenisation step. The study could also be further carried out at the molecular level through PCR analysis to quantify the difference in RNA yield.

## 4 Conclusion

The study highlights the benefits of a simple, quick-freezing method in maintaining RNA integrity without increasing costs or significantly prolonging sample processing time. Maintaining a cold temperature throughout homogenisation is key to the extraction of high-quality RNA. Our quick-freezing method ensures robustness and consistency in extracting high-quality RNA for gene expression analysis in mouse lung tissues, and further investigation could benefit nucleic acid studies.

## CRediT authorship contribution statement

**Khairunnisa Mohd Kamal:** Investigation, Methodology, Writing – original draft. **Ahmad Rohi Ghazali:** Supervision, Validation, Writing – review & editing. **Gayathri Thevi Selvarajah:** Supervision, Writing – review & editing. **Nurul Syakima Ab Mutalib:** Supervision, Validation, Writing – review & editing. **Nadiah Abu:** Supervision, Validation, Writing – review & editing. **Eng Wee Chua:** Methodology, Supervision, Validation, Writing – review & editing. **Siti Fathiah Masre:** Funding acquisition, Methodology, Supervision, Validation, Writing – review & editing.

## Declaration of competing interest

The authors declare that they have no known competing financial interests or personal relationships that could have appeared to influence the work reported in this paper.

## Data Availability

No data was used for the research described in the article.
